# Efficient removal of silver ions from wastewater via chelation with dithiooxamide-functionalized polymeric adsorbent

**DOI:** 10.1371/journal.pone.0338510

**Published:** 2025-12-31

**Authors:** Abdullah S. Al-Bogami, Abdullah Akhdhar, Dina A. Tolan, Mohamed H. Ismael, Emad A. Elshehy, Waleed A. El-Said

**Affiliations:** 1 Department of Chemistry, College of Science, University of Jeddah, Jeddah, Saudi Arabia; 2 Department of Chemistry, College of Science and Humanities, Prince Sattam bin Abdulaziz University, Alkharj, Saudi Arabia; 3 Department of Chemistry, Faculty of Science, Menoufia University, Shibin El-Kom, Egypt; 4 Nuclear Materials Authority, New-Cairo, Cairo, Egypt; Universiti Brunei Darussalam, BRUNEI DARUSSALAM

## Abstract

This study investigates Ag(I) adsorption on a dithiooxamide/glutaraldehyde resin (DTG-R) using both experimental and theoretical approaches. Characterization confirmed the resin’s porous structure and sulfur/nitrogen active sites. Batch experiments revealed high Langmuir capacity (27.2 mmol/g at 25°C), with kinetics following a pseudo-second-order model (*R*^*2*^ > 0.99), indicating chemisorption. Thermodynamic analysis showed endothermic (*ΔH°* = 121.25 kJ/mol), spontaneous adsorption (*ΔG°* = −12.8 to −17.3 kJ/mol), driven by entropy gains (*ΔS°* = 449.9 J/mol.K) from Ag(I) dehydration and polymer swelling. DFT calculations demonstrated preferential Ag(I) binding to deprotonated sulfur (S–Ag: 2.50–2.60 Å, bond order: 0.76–0.86) over nitrogen, with mononuclear complexes being most stable (*ΔE* = −175.6 kcal/mol). The resin exhibited high selectivity, reusability of 96% efficiency over five cycles, and optimal performance at pH 5.75. NBO analysis revealed charge transfer to Ag(I) (partial charge less than +1), while binding energy trends explained the observed temperature-dependent capacity. DTG-R combined high capacity, rapid kinetics, and molecular-level affinity for Ag(I) make it better than existing adsorbents for industrial wastewater remediation. This work bridges macroscopic adsorption properties with quantum-chemical mechanisms, offering a template for rational adsorbent design.

## 1. Introduction

The treatment of industrial and waste solutions containing high concentrations of toxic or valuable metal ions remains a critical research priority [1]. These solutions often contain trace levels of heavy metal ions, which can harm humans, animals, and plant life if released untreated [2]. Water contamination by heavy metals is growing in parallel with increased human activity and mining operations [3]. In nature, silver is typically associated with copper and lead deposits. It has widespread applications in jewelry, silverware, food and beverage processing, and metal alloys. Additionally, silver compounds are utilized in mirror production, photography, electroplating, catalysis, antimicrobial products, batteries, and ink manufacturing [4]. Given these uses, there is a pressing need to develop efficient, cost-effective, user-friendly, and sensitive techniques and materials for silver removal and recovery from industrial and waste streams.

The increasing demand for green and efficient extraction processes in separation technology has driven materials scientists to develop novel adsorbents for Ag(I) removal. Various materials, including activated carbon [5], natural clays [6], chelating resins [7], silicates [2,8,9], iron oxide [10], and biomass [11], have been investigated. Functionalized organic materials exhibit superior selectivity and regenerability compared to conventional options. These adsorbents leverage soft donor atoms (S, N, O) that preferentially bind soft acceptors like Ag(I), Au(III), and Hg(II) [7–12], with sulfur-containing polymers being particularly effective. Researchers have developed numerous functionalized adsorbents, including thiourea [7], amino-thiadiazole [13], bisthiourea [14], thiol/amine groups [8,15], dithiooxamide-formaldehyde [16], polythiazaalkane [17], dithiocarbamate [18], thiophene [19], and Amberlite XAD-16 resin [20] for enhanced silver recovery. The dithiooxamide/glutaraldehyde (DTO/GA) polymer has emerged as a highly effective chelating resin for the selective recovery of precious and heavy metal ions from aqueous solutions, a property primarily attributed to its dense population of sulfur and nitrogen donor atoms that act as potent Lewis basic sites for metal coordination [21]. This adsorbent is synthesized through a polycondensation reaction between dithiooxamide and glutaraldehyde, forming a robust, cross-linked network via Schiff base linkages (–N = C–) that is chemically stable and insoluble across a wide pH range [22]. The incorporation of the dithiooxamide monomer is crucial, as it embeds thiocarbonyl (C = S) and amine (N–H) groups into the polymer backbone, creating a microenvironment with a high affinity for soft Lewis acid metal ions like Ag(I), Au(III), and Pd(II), as predicted by the Hard-Soft Acid-Base (HSAB) principle [22]. The sulfur-rich DTO/GA polymer demonstrates exceptional selectivity for Ag(I) ions, exceeding 90% in multi-component metal solutions, with adsorption capacities reaching up to 2.8 mmol/g at an optimal pH of 5–6 [23]. This specific pH range is critical as it ensures the functional groups are sufficiently protonated to attract anionic complexes while remaining deprotonated enough for effective coordination, with adsorption kinetics that often follow a pseudo-second-order model, indicating chemisorption as the rate-limiting step [23,24]. Beyond adsorption, dithiooxamide serves as a key precursor for advanced nanocomposites and N/S-doped carbon catalysts. Synthesized through innovative, eco-friendly methods like hydrothermal carbonization, the N/S-doped carbon materials derived from DTO exhibit modulated electronic structures and enhanced conductivity [25]. These materials exhibit exceptional catalytic performance for environmental applications, including the catalytic reduction of organic pollutants such as methyl orange and the highly sensitive removal of toxins [26–28].

Building upon recent advancements in this field, we developed a novel sulfur- and nitrogen-containing polymeric adsorbent specifically designed for selective Ag(I) ion recovery. This research comprehensively examines the adsorption characteristics of Ag(I) ions using a chelating resin synthesized via straightforward copolymerization of dithiooxamide with glutaraldehyde. Our investigation encompassed: (1) determination of kinetic and thermodynamic adsorption parameters, (2) evaluation of resin regeneration and reusability potential, and (3) assessment of Ag(I) selectivity in multi-metal systems. Notably, this study represents significant methodological progress through quantum chemical modeling of both the polymer’s molecular orbitals (HOMO-LUMO) and [(DTG)-Ag] complex formation mechanism, providing fundamental insights into the adsorption process at the molecular level.

## 2. Materials and methods

### 2.1. Materials and characterization

Analytical-grade reagents were used as received. Dithiooxamide (Fluka, 98%) and glutaraldehyde (37% w/w) served as precursors, while AgNO₃ (Sigma-Aldrich, ≥ 99%) was the Ag(I) source. A 4.0 × 10 ⁻^2^  M Ag(I) stock solution in 1 M HNO₃ was stored in amber vessels to prevent photodecomposition. The synthesized chelating resin was comprehensively characterized using multiple analytical techniques: functional groups were identified by FT-IR spectroscopy (4000–400 cm ⁻^1^, KBr pellet); crystallinity was assessed via X-ray diffraction (5–80° 2θ, Cu Kα radiation); elemental composition and mapping were determined by EDX analysis; thermal stability was evaluated through thermogravimetric analysis (25–800°C, N₂ atmosphere); textural properties, including surface area, were derived from N₂ physisorption isotherms at 77 K; and electrical conductivity was measured using pressed pellets at 25 ± 1°C.

### 2.2. Synthesis of dithiooxamide/glutaraldehyde resin (DTG-R)

Dithiooxamide was first dissolved in 30 mL of 0.1 M NaOH solution, followed by addition of the glutaraldehyde solution under alkaline conditions (pH 10) at 80°C. The reaction mixture (3:1) was maintained at this temperature with continuous stirring until complete dissolution was achieved. Subsequently, the pH was carefully adjusted to 2 using a 10% HCl solution to initiate polymerization, with the reaction proceeding at 80°C for 6 h under constant agitation. The resulting polymeric condensate was then vacuum-filtered, sequentially washed with diluted acid, and then deionized water to remove unreacted monomers, oven-dried at 80°C, and mechanically ground to obtain uniform particles (<75 μm) suitable for adsorption studies [21].

### 2.3. Adsorption studies of Ag(I)

The adsorption performance of DTG-R for Ag(I) ions was evaluated through systematic batch experiments. Precisely 100 mg of finely ground adsorbent was added to 100 mL of Ag(I) solution (4 × 10 ⁻^2^ M) with pH adjusted between 1–8 using 0.1 M HNO₃ or NaOH to prevent Ag(OH) precipitation. The mixtures were agitated at 300 rpm (25°C) for 6 h to reach equilibrium, then vacuum-filtered through Whatman filter paper. Residual Ag(I) concentrations were quantified by Inductively Coupled Plasma Optical Emission Spectroscopy (ICP-OES). For adsorption isotherms, identical mass/volume ratios were tested across varying initial Ag(I) concentrations (1–50 mM) at the optimal pH, with 6 h of equilibration at 25 ± 1°C. The adsorption capacity at equilibrium (*qₑ,* mmol/g) was calculated using the following Equation 1 [8].


$$\;\;q_{e}=(C_{i}-C_{e})\text{V}/\text{m}\;\;\;$$ (1)

where *C₀* and *Cₑ* represent initial and equilibrium concentrations (mmol/L), *V* is solution volume (L), and m is adsorbent mass (g). Other experimental studies, such as uptake kinetics, sorption isotherms (25, 30, and 35°C), and the study of the effect of co-existing ions, were conducted under the same procedures. The DTG-R resin’s reusability was evaluated through column desorption studies. A 0.1 g resin bed (1.8 cm height × 0.1 cm diameter) was loaded with Ag(I) at optimal conditions (1 mL/min flow rate). Thiourea (0.5 M, acidified with HNO₃) effectively eluted adsorbed Ag(I), with complete regeneration achieved after DIW/alkali washing. Elution efficiency (*E%*) was calculated as Equation 2 [21].


$$\;\;E\%=\frac{q_{e\text{2}}}{q_{e\text{1}}}x\;\text{1}\text{0}\text{0}\;\;\;$$ (2)

where *q*_*e1*_ and *q*_*e2*_ are the total adsorption capacities of the first and second run, respectively. The adsorbent after desorption was refreshed and reused in sorption experiments, and the process was repeated several times. To assess real-world applicability, DTG-R was tested using industrial wastewater from a hospital. The multi-element sample, containing Ag(I) alongside other metals, was analyzed via ICP-OES to determine initial concentrations.

### 2.4. Computational methods

The quantum mechanical analysis was performed using density functional theory (DFT) [29,30] with the Gaussian 09 software package [31]. Geometry optimizations, frequency calculations, and molecular orbital generations were carried out at the B3LYP functional [32–34] with a 3–21 G(d) basis set. All calculations were carried out in the gas phase. Frequency analyses were performed on all optimized geometries at the same level of theory to confirm whether they correspond to minimal or transition states on the potential energy surface of the systems. The lack of negative eigenvalues in the force-constant matrices confirms that all identified stationary points correspond to minima. Computational results and FMO visualizations were generated using the ChemCraft program [35].

## 3. Results and discussion

### 3.1. Characterization of DTG-R adsorbent

**Fig 1:**
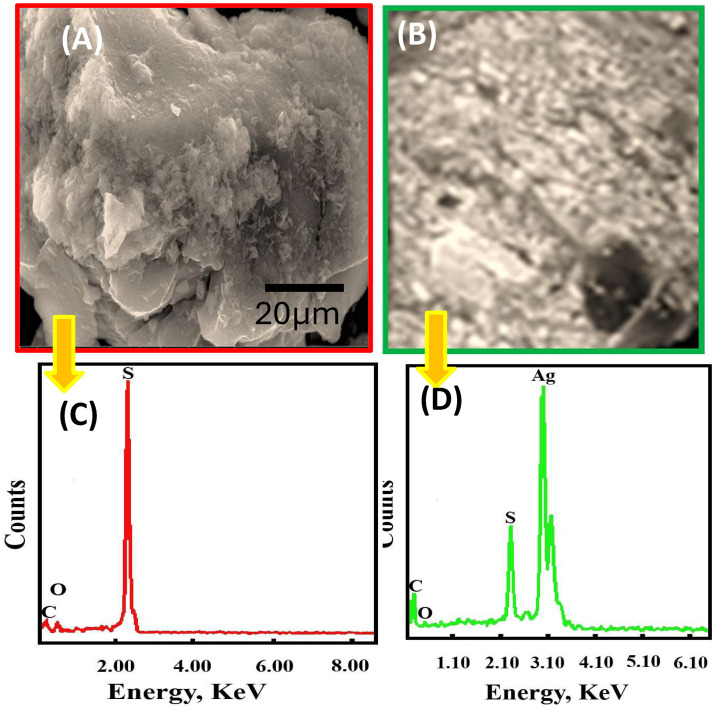
Comparative SEM images and EDX analysis of DTG-R resin. **(A)** SEM of free DTG-R, **(B)** SEM of DTG-R/Ag(I) resins, **(C)** EDX analysis for free DTG-R, and **(D)** EDX analysis for DTG-R/Ag(I) resins.

**Fig 2:**
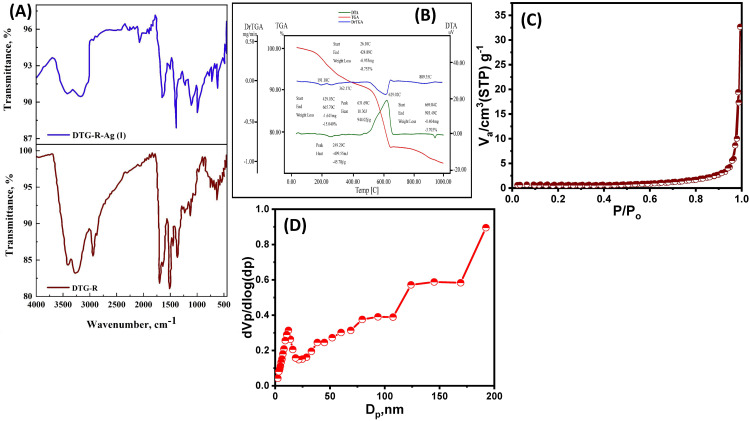
Characterization of DTG-R resin. **(A)** FTIR spectra before and after Ag(I) adsorption and **(B)** Thermal analysis (TGA, DTG, and DSC) of the Ag(I)-loaded DTG-R resin. **(C)** N_2_ adsorption/desorption isotherm, and **(D)** Pore size distribution of the DTG-R resin.

The structural and compositional properties of the synthesized dithiooxamide/glutaraldehyde resin (DTG-R) and its Ag(I)-loaded form were thoroughly characterized using multiple analytical techniques. SEM-EDX analysis further confirmed successful Ag(I) uptake, with distinct silver peaks appearing alongside the resin’s inherent sulfur signals. XRD studies demonstrated structural reorganization upon metal loading, with attenuation of sulfur-related peaks and emergence of new Ag-associated diffraction patterns (Fig 1). The enhanced electrical conductivity after Ag(I) adsorption suggested either complete surface coverage or possible Ag-Ag interactions [2,3]. FT-IR analysis of pristine DTG-R revealed characteristic functional group vibrations, including N-H stretching (3403–3235 cm ⁻^1^), C-H stretches (2936/2853 cm ⁻^1^), and distinctive peaks for C = C (1682 cm ⁻^1^), C-N (1494 cm ⁻^1^), and C = S (1118 cm ⁻^1^), confirming successful polymerization [21]. Upon Ag(I) adsorption, significant spectral shifts were observed, particularly in the C-N and C = S regions (shifting downward by 99 cm ⁻^1^), along with the appearance of a new S-Ag vibration at 715–720 cm ⁻^1^, clearly indicating coordination between Ag(I) and the resin’s nitrogen/sulfur sites (Fig 2A).

Thermogravimetric analysis showed improved thermal stability of the Ag-loaded resin, with only 27% mass loss compared to 50.25% for the pristine polymer, while the remaining 73% residue quantitatively matched the adsorbed silver content. These comprehensive characterization results collectively validate DTG-R’s effectiveness as a high-capacity adsorbent and provide clear evidence of the strong coordination between Ag(I) ions and the resin’s functional groups (Fig 2B). The thermal stability and structural integrity of the Ag-loaded resin further support its potential for practical applications in silver recovery processes. The N₂ adsorption-desorption analysis indicates a mesoporous material with a BET surface area of 14.6 m^2^/g, a pore volume of 0.154 cm^3^/g, and an average pore diameter of 45.2 nm (Fig 2C and 2D).

### 3.2. Silver ions adsorption using the batch method

#### 3.2.1. Effect of solution pH.

**Fig 3:**
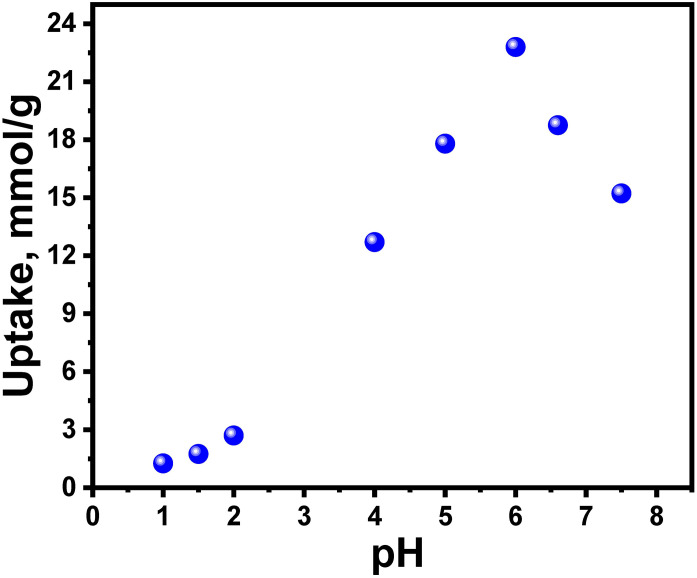
Effect of equilibrium pH on the adsorption of Ag(I) ions on DTG-R; initial concentration of Ag(I) 4 × 10^−2^ M, equilibrium time of Ag(I) 6 h, DTG-R weight 100 mg, solution volume 50 mL, at 25°C.


The adsorption behavior of Ag(I) ions on the synthesized DTG-R resin was systematically investigated through batch experiments to elucidate the pH-dependent uptake mechanism. The process exhibited maximum adsorption capacity at equilibrium (22.8 mmol/g at 25°C) at pH 5.75 (Fig 3), with three distinct pH-dependent regions observed: (1) In strongly acidic conditions (pH < 2), minimal adsorption occurred due to competitive protonation of active sites (RNH₂ + H⁺ → RNH₃⁺) and suppression of thiol group dissociation (RSH → RS⁻ + H⁺), following the equilibrium described in Equation 3 [21–23]. (2) The optimal pH range (4.6–6.0) facilitated coordination through both deprotonated thiolate (-S⁻) and neutral amine (-NH₂) groups, as evidenced by the significant pH decrease (5.75 to 2.2) during adsorption, confirming proton release via ion-exchange (Equation 4). (3) In alkaline media (pH > 6), apparent removal efficiencies were artificially enhanced by Ag(OH) precipitation (pH 7–8), while above pH 8, decreased uptake resulted from the formation of soluble Ag(OH)₂ ⁻ complexes that experienced electrostatic repulsion with the negatively charged resin surface [2,8].The adsorption mechanism follows Hard-Soft Acid-Base (HSAB) principles, where soft Lewis basic sites (S, N) preferentially coordinate with soft Ag⁺ cations, forming stable [Ag(L)]⁺ complexes [13]. Electrophoretic mobility measurements confirmed these interactions through changes in surface charge characteristics during adsorption. This pH-dependent behavior demonstrates DTG-R’s superior selectivity for Ag(I) recovery compared to conventional adsorbents, with the optimal pH window balancing site availability and metal speciation.

$$\text{DTG}-\text{R}----\text{N}{\text{H}}^{+}\;{\text{X}}^{+}+\;\text{A}{\text{g}}^{+}\;\to \;\text{DTG}-\text{R}---\text{NA}{\text{g}}^{+}\;{\text{X}}^{-}+{\text{H}}^{+}$$ (3)

$$\text{DTG}-\text{R}\;----\text{SH}+{\text{X}}^{-}+\;\text{A}{\text{g}}^{+}\;\to \;\text{DTG}-\text{R}---\text{SA}{\text{g}}^{+}\;{\text{X}}^{-}+{\text{H}}^{+}$$ (4)

The adsorption of Ag(I) onto DTG-R resin exhibited strong pH dependence, achieving optimal uptake of 22.8 mmol/g at pH 5.75, significantly higher than conventional adsorbents like activated carbon (2–5 mmol/g) and chitosan derivatives (8–12 mmol/g) under similar conditions [36]. The distinct pH decreases from 5.75 to 2.2 during adsorption confirmed an ion-exchange mechanism, where Ag(I) displaced protons from -SH and -NH₂ groups [13]. Below pH 4, capacity decreased sharply to 3.0 mmol/g at pH 2 due to proton competition. Above pH 6, silver hydroxide formation artificially inflated removal efficiencies. The pH of 4.6–6.0 maximized coordination through deprotonated S/N donors, following HSAB principles, with kinetics (k₂ = 8.3 × 10 ⁻ ⁴ g/mmol·min) 2–3 times faster than thiol-functionalized mesoporous silicas [8].

#### 3.2.2. Adsorption kinetics.

**Fig 4:**
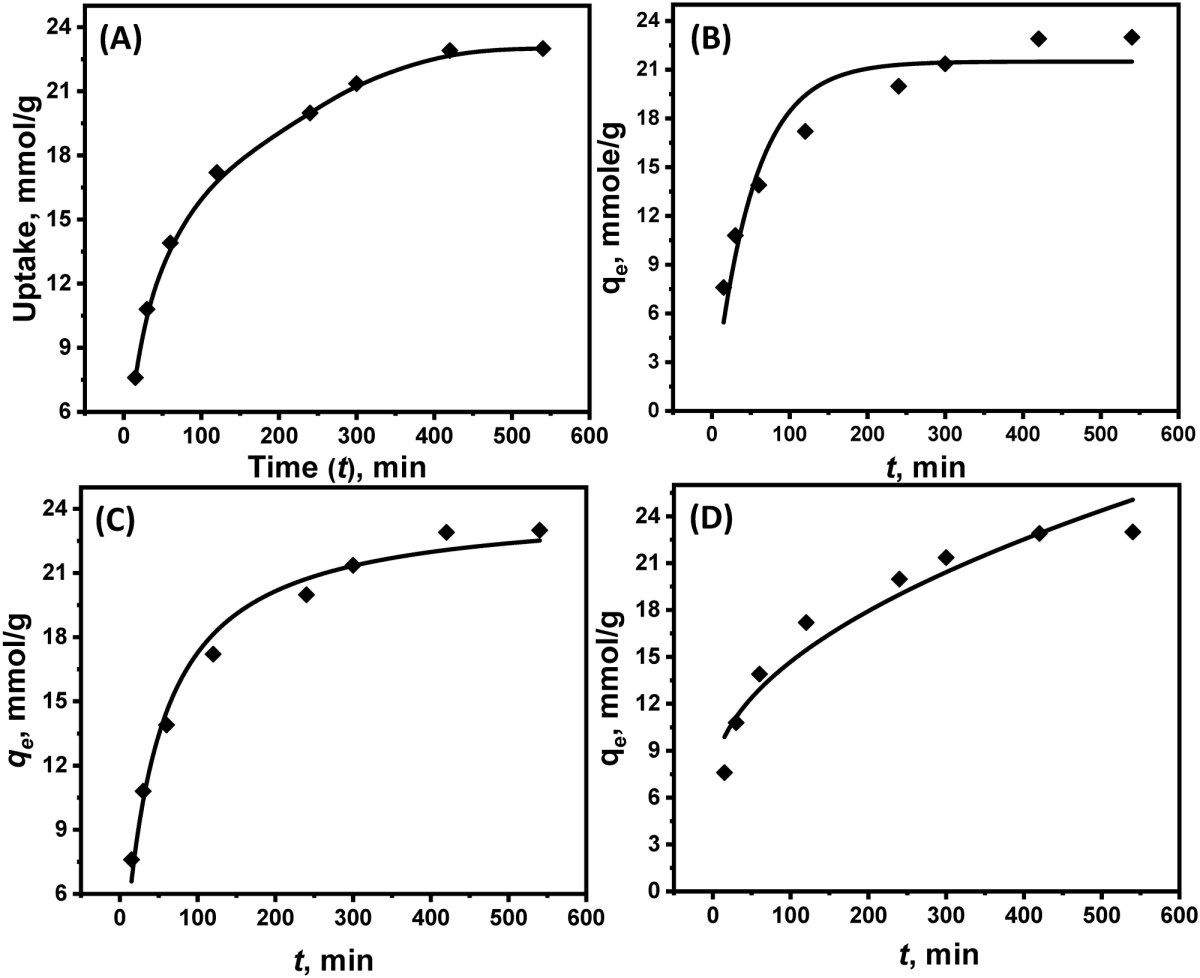
Kinetic analysis and modeling of Ag(I) adsorption on DTG-R resin. **(A)** Effect of contact time on the adsorption of Ag(I) (pH = 5.75 with initial concentration 4 × 10^−2^ M) on DTG-R from a single ion solution; DTG-R weight 100 mg, solution volume 50 mL at 25°C. Ag(I) uptake kinetics with fitted lines using the PFOR **(B)** and PSOR **(C)** models. **(D)** The Weber and Morris intraparticle diffusion model.

The adsorption kinetics of Ag(I) ions onto the multi-thiol/amine-functionalized DTG-R resin were investigated under optimized conditions (pH 5.75, initial concentration 4 × 10 ⁻^2^ M, 25°C). As shown in Fig 4A, the adsorbent exhibited a rapid initial uptake, achieving 60% of total capacity within the first 60 min, followed by gradual equilibration over 6 h, reaching a final capacity of 22.5 mmol/g. The prolonged equilibration suggests a hybrid mechanism involving both surface adsorption and intraparticle diffusion, with the latter being hindered by pore penetration and electric double layer (EDL) effects.

The kinetics of the adsorption process are further studied using several models. We first used the conventional pseudo-first-order (PFOR) and pseudo-second-order (PSOR) rate equations to fit the kinetic profile (Equations 5 and 6) [37,38].


$$q_{t}=q_{e}\;\;(\text{1}-e^{-kt})\;\;\;$$ (5)

$$q_{t}=\frac{k_{\text{2}}q_{e}^{\text{2}}t}{\text{1}+k_{\text{2}}q_{e}t}$$ (6)

where *k₁* (min ⁻^1^) and *k₂* (g/mmol·min) represent their respective rate constants, and *qₜ* (mmol/g) indicates the time-dependent Ag(I) ions uptake capacity. Nonlinear regression analysis revealed comparable determination coefficients (*R*^2^) for both models, with PFOR showing marginally better agreement (Fig 4). Kinetic modeling revealed the process followed pseudo-second-order (PSOR) kinetics (R^2^ = 0.983, *k₂* = 1.0 × 10 ⁻^3^ g/mmol.min) more closely than pseudo-first-order (PFOR) (R^2^ = 0.923, *k₁* = 1.9 × 10 ⁻^3^ min^-1^), indicating chemisorption dominated by coordination bonding between Ag(I) and the resin’s thiol/amine groups. However, the marginal PFOR agreement suggested additional physical adsorption contributions. Additionally, the PFOR-predicted equilibrium capacity (21.5 mmol/g) closely matched experimental values, while PSOR overestimated uptake (24.2 mmol/g). The determined *R*^*2*^ value of the PSOR (0.983) is higher than that of PFOR (0.923) for Ag(I) ions, suggesting that Ag(I) ions’ sorption rate into the DTG-R is better modeled with the second-order model. Since PFOR is supposed to fit profiles controlled by physical sorption, while the PSOR is usually associated with chemical sorption, the results suggest a more chemically controlled process. This ambiguity suggests a hybrid adsorption mechanism, as PFOR typically describes physical sorption, whereas PSOR reflects chemisorption. Further analysis of the kinetic profiles (Fig 4B and 4C) identified a three-stage adsorption process: (i) rapid initial Ag(I) ions transport to the adsorbent surface. (ii) intraparticle diffusion via gradual pore penetration through the DTG-R structure, and (iii) formation of stable [DTG-R → Ag(I)]ⁿ⁺ complexes via coordination bonds

The Weber and Morris intraparticle diffusion model was employed to further elucidate the Ag(I) adsorption process, Equation 7 [39,40]:


$$q_{t\;}\;=x+\;k_{i\;}t^{\text{0}.\text{5}}$$ (7)

where *x* represents boundary layer thickness and *kᵢ* denotes the intraparticle diffusion rate constant (mmol/g.min⁰·⁵). The nonlinear fitting results (Fig 4D) demonstrate that Ag(I) uptake by the DTG-R adsorbent is predominantly governed by intraparticle diffusion, as evidenced by the strong correlation between the experimental data and the model. Using Weber-Morris intraparticle diffusion model (kᵢ = 0.784 mmol.g ⁻^1^.min ⁻ ⁰.⁵, x = 6.84, R^2^ = 0.933) confirmed a three-stage mechanism: (1) rapid surface adsorption (boundary layer diffusion, evidenced by non-zero x), (2) gradual intraparticle diffusion through resin pores, and (3) final equilibration via stable [DTG-R → Ag(I)] complex formation. The high correlation underscored intraparticle diffusion as the rate-controlling step, consistent with the resin’s porous structure that balanced Ag(I) transport with sufficient dwell time for chemisorption. The kinetic profiles aligned with the resin’s design, showing initial fast binding to surface functional groups followed by slower matrix penetration and eventual strong coordination with multiple donor sites.

These results demonstrate the DTG-R resin’s superior performance over conventional adsorbents, combining high capacity (22.5 mmol/g) with practical kinetics. The multistep adsorption process involving both rapid surface binding and slower intraparticle diffusion highlights the importance of the resin’s tailored porosity and abundant thiol/amine groups for efficient Ag(I) recovery. The PSOR dominance confirms the chemisorption mechanism, while the residual PFOR contribution reflects the complex interplay between physical and chemical adsorption in this system.

#### 3.2.3. Influence of Ag(I) concentrations on adsorption isotherms.

**Fig 5:**
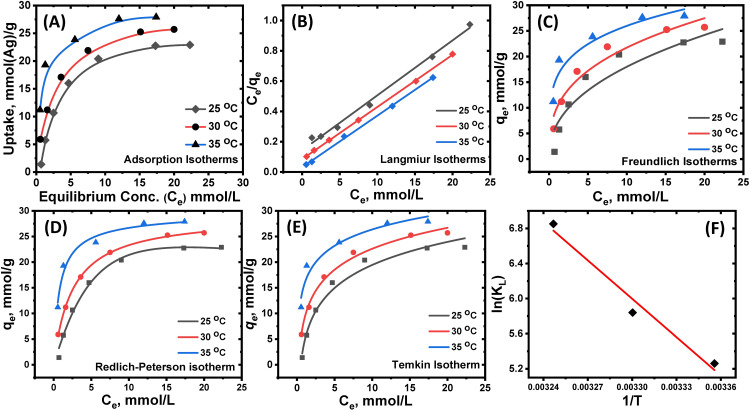
Adsorption isotherms, model fitting, and thermodynamic study of Ag(I) on DTG-R. **(A)** Adsorption isotherms of Ag(I) (pH = 5.75, time 6 h) on DTG-R sorbent from a single ion solution; DTG-R weight 100 mg, solution volume 50 mL at different temperatures (25, 30, and 35°C). **(B)** Linear Langmuir isotherms, **(C)** Nonlinear Freundlich isotherms, **(D)** Nonlinear Redlich-Peterson model isotherms, and **(E)** Nonlinear Temkin model isotherms. **(F)** Van’t Hoff plot for the adsorption of Ag(I) on DTG-R sorbent.

The equilibrium adsorption behavior of Ag(I) onto the DTG-R polymeric adsorbent was systematically investigated through detailed isotherm analysis at three different temperatures (25, 30, and 35°C), as shown in Fig 5A. The experimental results demonstrate a clear temperature-dependent enhancement in adsorption capacity with measured *q*_*e*_ values increasing from 23.0 mmol/g at 25°C to 27.2 mmol/g at 35°C. This positive correlation between adsorption capacity and temperature indicates the fundamentally endothermic nature of the adsorption process, which can be attributed to several interrelated mechanisms operating at the molecular level.

The observed temperature effect primarily stems from three distinct but complementary phenomena: First, thermal activation induces swelling of the polymer matrix, which expands the pore structure and enhances accessibility to internal binding sites while simultaneously facilitating the diffusion of Ag(I) through the adsorbent’s microstructure. Second, elevated temperatures promote partial dehydration of both the adsorbent’s functional groups (-SH, -NH) and the hydrated Ag(I) [Ag(H₂O)ₙ]⁺, reducing steric hindrance and enabling stronger coordination interactions between the metal ions and binding sites. Third, increased thermal energy boosts the kinetic energy of Ag(I) in solution, resulting in higher collision frequency with adsorption sites and greater probability of successful binding events [2,3].

The adsorption isotherms exhibit characteristic Langmuir Type I behavior, featuring a sharp initial increase in uptake at low concentrations followed by a distinct plateau at higher concentrations. This profile suggests monolayer adsorption occurring on energetically homogeneous sites, with saturation being reached when complete surface coverage is achieved. To fully characterize this behavior, four different isotherm models were applied and their parameters rigorously analyzed. The abrupt increase in the adsorption capacity at low concentration ranges is characteristic of the adsorption on chelating-adsorbent as classified by type-I of Langmuir’s classification, according to the following equation 8 [41].


$$\frac{{\text{C}}_{\text{e}}}{q_{e}}=\frac{{\text{C}}_{\text{e}}}{{\text{q}}_{\text{L}}}+\frac{\text{1}}{{\text{K}}_{\text{L}}{\text{q}}_{\text{L}}}\;$$ (8)

where *C*_*e*_ is the equilibrium concentration of silver ions in solution (mmol/L), *q*_*e*_ is the adsorbed value of silver ions at equilibrium (mmol/g), *q*_*L*_ is the theoretical maximum adsorption capacity (mmol/g), and *K*_*L*_ is the Langmuir binding constant, which is related to the energy of adsorption (L/mmol). Plotting *Ce*/*q*_*e*_ against *Ce* gives a straight line with slope and intercept equal to 1/*q*_*L*_ and 1/*K*_*L*_ *q*_L_, respectively, Fig 5B. The values of *q*_*L*_, binding constant *K*_*L,*_ and determination coefficients *R*^*2*^ at different temperatures were obtained and reported in Table 1. The Langmuir model provided excellent fits to the experimental data (R^2^ > 0.99), yielding maximum theoretical capacities (*q*_*L*_) of 27.5, 28.9, and 29.3 mmol/g at 25, 30, and 35°C, respectively. These values show remarkable agreement (±4%) with the experimentally measured capacities, validating the model’s applicability. The binding constant *K*_*L*_ exhibited an interesting non-monotonic temperature dependence, increasing from 0.25 L/mol at 25°C to a maximum of 41.0 L/mol at 30°C before decreasing to 1.1 L/mol at 35°C. This peak at 30°C may indicate an optimal temperature for adsorption affinity beyond which structural changes in the polymer or alterations in the hydration sphere of Ag(I) could slightly reduce binding strength [8]. The separation factor *R*_*L*_, calculated using Equation 9, consistently fell between 0 and 1 across all tested conditions, confirming thermodynamically favorable adsorption [15]. Notably, *R*_*L*_ values approached zero at higher temperatures, suggesting increasingly irreversible binding behavior under these conditions.

**Table 1:** Adsorption isotherm parameters for Ag(I) adsorption on DTG-R at different temperatures.

Isotherm Model	Parameter	25°C	30°C	35°C
**Langmuir**	*q*_*L*_ (mmol/g)	27.5	28.9	29.3
	*K*_*L*_ (L/mol)	0.25	41.0	1.10
	*R* ^2^	0.9936	0.9997	0.9984
	*R*^*2*^ *Adj.*	0.9920	0.9996	0.9989
**Freundlich**	*K*_*F*_ (L^1/n^.mmol ^(1–1/n)^)/g	6.73	9.98	15.9
	*n*	2.3	2.9	4.5
	*R* ^2^	0.8908	0.9457	0.9125
	*R*^*2*^ *Adj.*	0.8690	0.9322	0.8833
**Redlich-Peterson**	*K*_*RP*_ (L/g)	4.75	11.7	40.6
	*a* (L/mmol)	0.052	0.4	1.5
	*β*	1.37	0.99	0.97
	*R* ^2^	0.9906	0.9993	0.9769
	*R*^*2*^ *Adj.*	0.9860	0.9989	0.9539
**Temkin**	*A* (L/g)	2.0	4.6	30.9
	*B* (J/mol)	6.48	5.9	4.6
	*R* ^2^	0.9799	0.9917	0.9487
	*R*^*2*^ *Adj.*	0.9759	0.9896	0.9317


$$R_{L}\;=\frac{\text{1}}{\text{1}+K_{L}C_{o}}\;\;\;$$ (9)

Complementary analysis using the Freundlich model revealed important information about surface heterogeneity and adsorption intensity (Equation 10) [42].


$$q_{e}=k_{F}c_{e}^{\text{1}/n}$$ (10)

The capacity parameter *K*_*F*_ increased steadily from 6.73 to 15.9 (L^1^/^n^ mmol^(1^ ⁻ ^1^/^n)^/g) with rising temperature, while the intensity parameter *n* increased from 2.3 to 4.5. These *n* values, all greater than 1, indicate favorable adsorption conditions, with the increasing trend suggesting reduced surface heterogeneity at higher temperatures. The Freundlich model’s slightly lower *R*^2^ values (0.890–0.945) compared to Langmuir imply that while surface heterogeneity exists, it does not dominate the adsorption behavior.

The Redlich-Peterson model, which incorporates features of both Langmuir and the Freundlich isotherms, provided additional insights through its three parameters (Equation 11).


$$q_{e}=\frac{K_{RP}\;C_{e}}{\text{1}+a\;C_{e}^{n}}$$ (11)

The *K*_*RP*_ values showed a dramatic increase from 4.75 to 40.6 L/g with temperature, while the exponent *β* approached unity (decreasing from 1.37 to 0.97), indicating the system becomes more Langmuir-like at elevated temperatures. The parameter *a*, related to adsorption energy, increased from 0.052 to 1.5 L/mmol, reflecting stronger binding at higher temperatures. The excellent fits (*R*^*2*^ = 0.990–0.998) confirm the model’s ability to capture subtle aspects of the adsorption process.

Thermodynamic analysis through the Temkin model yielded particularly valuable information about the heat of adsorption (Equation 12).


$$q_{e}\;=\frac{RT}{B}ln\left(\;A\;C_{e}\right)\;\;\;\;\;$$ (12)

The binding constant *A* increased substantially from 2.0 to 30.9 L/g with temperature, while the heat of adsorption parameter *B* decreased from 6.48 to 4.6 J/mol. This inverse relationship is characteristic of systems where adsorption becomes more favorable entropically at higher temperatures, even as the heat of adsorption per site decreases. The high binding energies (>40 kJ/mol calculated from *B* values) provide strong evidence for chemisorption as the dominant mechanism (Table 1). Comprehensive isotherm analysis collectively demonstrates that Ag(I) adsorption on DTG-R occurs primarily through chemical complexation with sulfur and nitrogen functional groups, with temperature playing a crucial role in modulating both capacity and affinity.

The experimental and modeled adsorption data demonstrate excellent agreement, with the measured capacities (*q*_*e*_ = 22.8, 25.3, and 27.2 mmol/g at 25, 30, and 35°C, respectively) approaching the Langmuir-predicted *q*_*L*_ values (27.5–29.3 mmol/g), confirming the model’s validity for this system. The temperature-dependent increase in both experimental and theoretical capacities correlates with rising Freundlich *K*_*F*_ values (6.73 to 15.9) and Langmuir *K*_*L*_ trends, peaking at 30°C (41 L/mol) before decreasing at 35°C, suggesting an optimal binding temperature exists. The consistently high *R*^2^ values for Langmuir fits (0.993–0.999) versus Freundlich (0.890–0.945) and Temkin (0.948–0.991) models indicate monolayer adsorption dominates, though the Freundlich *n* values (2.3–4.5) and Temkin parameter variations reveal additional surface heterogeneity and coverage-dependent energetics (Table 1). The decreasing Temkin constant *B* values (6.48 to 4.6 J/mol) with temperature further support that adsorption becomes more favorable at higher temperatures despite reduced interaction energies per site, consistent with the endothermic nature of the process demonstrated by the capacity increases. These comprehensive isotherm analyses collectively verify that Ag(I) adsorption on DTG-R occurs primarily through chemisorption with temperature-enhanced accessibility of active sites.

The adsorption performance of the developed DTG-R resin (22.8 mmol/g) significantly surpasses most reported adsorbents for silver recovery. Among thiourea-based polymers, capacities range from 0.95 mmol/g for melamine-formaldehyde-thiourea [43] to 13.2 mmol/g for thiourea-formaldehyde resins [16], with bisthiourea variants typically showing intermediate performance (2.1–8.1 mmol/g) [7,14,44]. Amino-functionalized resins demonstrate more modest capacities (0.5–1.2 mmol/g) [45,46], while clay minerals exhibit particularly low uptake (0.019–0.68 mmol/g) [47,48]. Magnetic nanocomposites show improved but variable performance (1.17–1.32 mmol/g) [49,50], with chitosan-thiourea composites lacking reported quantitative data. Biosorbents present a wide capacity range from spent coffee grounds biochar (0.45 mmol/g) [51] to exceptional performance by Sargassum algae (8.67 mmol/g) [52]. Specialty adsorbents show particularly interesting results with molybdenum oxide mixed-valence materials achieving a remarkable 24.2 mmol/g capacity [53], though polymer analogs like PDMTD are less effective (1.19 mmol/g) [54]. The dithiooxamide-formaldehyde resin reported by Çelik et al. exhibits very high capacity of 30.86 mmol/g, attributed to its specific affinity for Ag(I) over other base metals [55]. Similarly, Li et al. documented a sponge-like thiourea-formaldehyde resin with a high adsorption capacity of 23 mmol g ⁻^1^ for silver nanoparticles, facilitated by its hierarchically porous structure and synergistic electrostatic and metal-ligand interactions [56]. Other notable adsorbents include flower-like thiourea/formaldehyde microspheres with a capacity of 18.17 mmol/g [57], a grafted thiourea resin with 5.58 mmol g ⁻^1^ [58], and poly(3-mercaptopropylsilsesquioxane) microspheres reaching 10.6 mmol g ⁻^1^ [59]. The adsorption is generally optimal in acidic conditions (pH ~ 2–6), follows the Langmuir isotherm and pseudo-second-order kinetics, and is driven primarily by chelation mechanisms involving S and N atoms. These findings highlight the critical role of tailored functionalization and morphology in enhancing silver recovery for environmental and hydrometallurgical applications. The DTG-R resin’s 22.8 mmol/g capacity not only exceeds nearly all polymeric and biological adsorbents but also competes favorably with the highest-performing specialty materials, while offering advantages in terms of selectivity, regenerability, and cost-effectiveness compared to inorganic adsorbents like molybdenum oxides.

#### 3.2.4. Thermodynamic studies on the adsorption of Ag(I) on DTG-R resin.

The temperature-dependent Langmuir binding constants (*K*_*L*_) were analyzed using the Van’t Hoff equation [10,12] to determine the thermodynamic parameters of the adsorption process. This approach allowed for quantification of the enthalpy change (*ΔH°*) through the linear relationship between *ln(K*_*L*_*)* and *1/T*, while the intercept provided information about the entropy change (*ΔS°*). The Van’t Hoff analysis confirmed the endothermic nature of Ag(I) adsorption onto DTG-R, as evidenced by the positive *ΔH°* values, and revealed favorable entropy changes contributing to the spontaneity of the process. The strong temperature dependence of *K*_*L*_ values further supported the chemisorption mechanism previously indicated by the high Temkin heats of adsorption (Equation 13) [15].


$$lnK_{L}=\frac{-\Delta H\circ }{RT}+\frac{\Delta S\circ }{R}\;\;\;\;\;\;\;\;\;\;\;\;\;\;\;\;\;\;\;\;\;\;\;\;\;\;\;\;\;\;\;\;\;\;\;\;\;\;$$ (13)

Thermodynamic analysis was performed using the Van’t Hoff equation, where *R* represents the universal gas constant (8.314 J mol ⁻^1^ K ⁻^1^) and *T* is the absolute temperature (K). The linear Van’t Hoff plot of *ln K*_*L*_ versus *1/T* (Fig 5) yielded thermodynamic parameters from its slope and intercept. Additionally, Gibbs free energy change (*ΔG°*) was determined using the fundamental relationship (Equation 14) [15].


ΔG∘ = ΔH∘ − TΔS∘ (14)

The negative *ΔG°* values confirmed the spontaneous nature of the adsorption process, while the positive *ΔH°* indicated its endothermic character. The positive *ΔS°* suggested increased randomness at the solid-liquid interface during adsorption, consistent with the observed temperature-dependent enhancement of Ag(I) uptake.

The thermodynamic analysis of Ag(I) adsorption on DTG-R resin revealed an endothermic yet spontaneous process, evidenced by a positive enthalpy change (*ΔH°* = 121.25 kJ/mol) and negative Gibbs free energy (*ΔG°* = −12.8 to −17.3 kJ/mol at 25–35°C). The large entropy gain (ΔS° = +449.9 J/mol·K) indicates significant structural reorganization, including dehydration of hydrated Ag(I) and swelling of the polymer matrix, which enhances accessibility to binding sites. The endothermic nature explains the observed temperature-dependent increase in adsorption capacity, where higher temperatures (25–35°C) strengthened Ag(I) uptake due to improved ion mobility and stronger coordination with sulfur and nitrogen functional groups.

### 3.3. DFT studies

**Fig 6:**
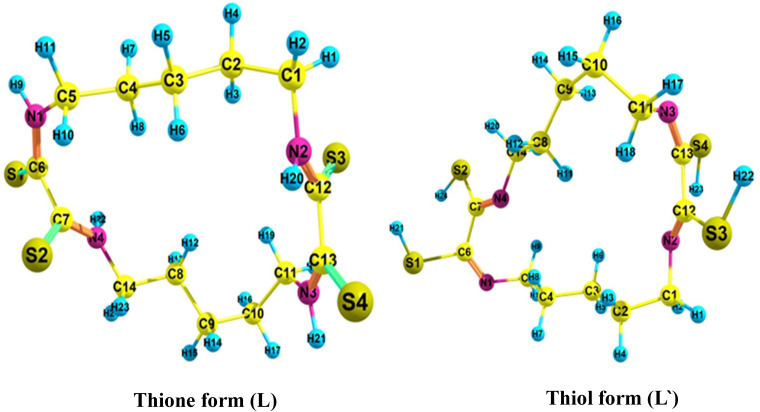
Optimized structures of the DTG-R ligand tautomer.

**Fig 7:**
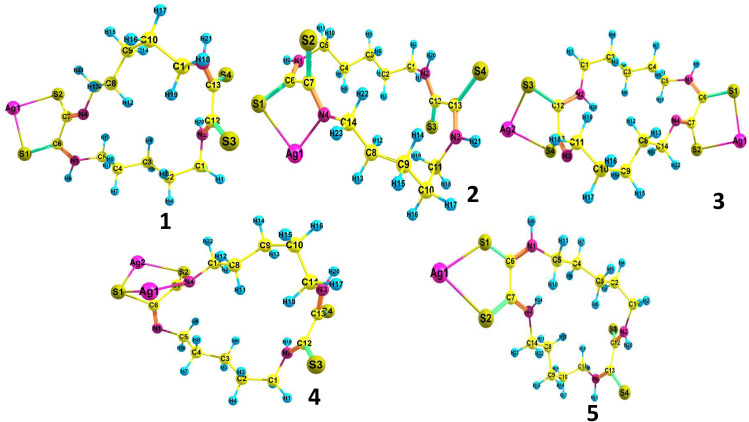
Optimized structures of the Ag (I) suggested complexes (1-5) at B3LYP/3-21 G(d).

As demonstrated experimentally, the resin (DTG-R) exhibits high and selective sorption capacity for silver ions. It is essential to gain a more comprehensive understanding of the mechanisms underlying the sorption processes. The DFT method is a valuable tool that offers deeper insight into the nature of interactions occurring during adsorption [60,61]. Theoretical simulations of silver complexes were carried out to gain insight into the binding interactions of silver ions with the developed resin. Five structures of silver complexes and the DTG-R ligand were optimized at the DFT/B3LYP level using the Gaussian 09 program. The optimized geometries of the ligand tautomers and their corresponding complexes are shown in Figs 6 and 7, with detailed geometric parameters for some selected complexes provided in S1 Table in S1 File. The IR frequencies were calculated and verified to contain no imaginary frequencies, indicating that the investigated systems represent minima on their potential energy surface.

The thione and thiol forms of the ligand were optimized in the gas phase at the DFT/B3LYP level of calculation. The calculations indicated that the thione form (**L**) is more stable than the thiol form (**L**`) by 72 kcal/mol, which aligns with the experimental observations. To analyze the charge distribution on the ligand, Natural Bond Orbital (NBO charge) analysis of the ligand moiety was conducted. The NPA charges for selected atoms in the ligand and its complexes are presented in S2 Table in S1 File. While the negative charge on the nitrogen atoms is higher than that of sulfur atoms, silver (Ag) has a strong affinity for sulfur. This affinity is a result of the interaction between the soft base nature of silver ions (Ag⁺) and the soft acid nature of sulfur atoms. In the mononuclear complex 1, the silver ion binds to the deprotonated ligand through two sulfur atoms, one deprotonated sulfur atom by an ionic bond, while it binds to the other sulfur atom by a coordination bond, forming a five-membered chelate ring. In the mononuclear complex 2, the DTG-R coordinates to Ag (I) by the deprotonated thiol sulfur atom and one amine nitrogen atom, forming a five-membered chelate ring, respectively. The bond distances S-Ag in complex 1 are 2.57 and 2.50 Å, while NAO (Natural Atomic Orbital) bond orders are 0.76 and 0.86. For complex 2, the bond distances S-Ag and N-Ag are 2.60 Å and 2.22 Å, with NAO bond orders of 0.35 and 2.28, respectively. These results indicate that Ag(I) exhibits a stronger interaction with the deprotonated sulfur atom compared to the amine nitrogen atom. On the other hand, in the binuclear complex 3, each Ag(I) center interacts with two sulfur atoms, similar to complex 1, forming an ionic bond with a deprotonated sulfur atom and a coordination bond with the second sulfur atom, while one of the Ag (I) atoms in the binuclear complex 4 binds to two sulfur atoms. The other binds to one nitrogen and one sulfur atom, forming two fused five-membered rings. The NBO partial charges of Ag(I) in all the complexes are lower than +1, indicating charge transfer from the ligands to the Ag(I) centers during coordination. Among these, complexes 1 and 3 exhibit the lowest positive charges on Ag(I), confirming a stronger interaction with deprotonated sulfur atoms compared to amine nitrogen atoms or protonated sulfur atoms.

Binding energy (B.E.) serves as a valuable parameter to assess the thermodynamic favorability of the formed adsorbed system. It is defined as the difference in energy between the optimized structures of the final state (after adsorption) and the initial state (before adsorption). As shown in Table 2, the binding energies of the studied complexes are all negative, indicating that the complex reactions are thermodynamically favorable. The trend in binding energy follows the order: complex 1 < complex 2 < complex 3 < complex 4 < complex 5. The binding energies of the mononuclear complexes are more negative than those of the binuclear ones, which may be attributed to a decrease in the ligand’s nucleophilicity upon coordination with a silver atom, thereby reducing its tendency to bind additional silver ions. Nevertheless, the negative binding energies of the binuclear complexes still indicate that the ligand retains sufficient donor ability to coordinate more than one silver ion per molecule. The binding energy of complex 1 is more negative than that of complex 2, with a small difference of 2.75 kcal/mol. To evaluate the role of thiol deprotonation in complexation with Ag(I), we calculated the binding energy of the cationic complex 5, which lacks thiol deprotonation and serves as an analog of complex 1. The results show that the binding energy of complex 1, where Ag(I) coordinates to a deprotonated sulfur atom, is significantly more negative (~ −175 kcal/mol) than that of complex 5 (~ −0.11 kcal/mol). This stark contrast indicates that deprotonation of the DTG-R ligand is critical for effective coordination with Ag(I), thereby supporting our experimental observation that chelation occurs through the deprotonated thiol form of the ligand.

**Table 2:** Calculated binding energies (kcal/mol) for the studied complexes in the gas phase.

Complexes	∆E_0_	∆E_298_	∆H_298_	∆G_298_
**1**	−175.64	−175.42	−176.02	−167.03
**2**	−173.06	−172.93	−173.53	−163.83
**3**	−12.74	−12.12	−13.31	2.60
**5**	−20.50	−20.08	−21.27	−4.10
**4**	−0.114	−0.113	−0.114	−0.10

The proposed adsorption mechanism involves the coordination of silver ions to the sulfur atoms of the DTG-R ligand, after thiol group deprotonation. Following this initial interaction, the ligand exhibits sufficient donor capacity to bind multiple silver ions per molecule, indicating the potential for multidentate coordination behavior.

#### 3.3.1. Elution and regeneration performance of the resin.

In removal/recovery purposes, one of the most important factors is the use of materials that exhibit high performance, reproducibility, durability, and recyclability. The effective elution of the studied chelating adsorbent was found feasible using a solution of 0.5 M thiourea acidified with 0.5 M HNO_3_ for the recovery of Ag(I). The adsorbent was reused effectively up to the 5^th^ cycle of regeneration and reuse, after which a subtle decrease in adsorption efficiency was observed. Elution efficiency was found to be 96.0% for the recovery Ag(I). The resin exhibited outstanding reusability over five consecutive adsorption-desorption cycles, maintaining high performance with only a marginal reduction in efficiency beyond the fifth cycle.

## 4. Conclusions

This study successfully developed DTG-R, an adsorbent achieving exceptional Ag(I) uptake with an experimental capacity is 22.8 mmol/g at 25°C and maintaining 96% elution efficiency over five cycles. The spontaneous, endothermic adsorption followed pseudo-second-order kinetics, with a high enthalpy change (ΔH° = 121.25 kJ/mol) confirming chemisorption. DFT calculations quantitatively validated the mechanism, revealing preferential Ag(I) binding to deprotonated sulfur sites in stable mononuclear complexes with a binding energy of −175.6 kcal/mol. By integrating macroscopic experiments with quantum-chemical modeling, this work provides a high-performance material for silver recovery and a rational design blueprint for targeted adsorbents, demonstrating significant potential for application in e-waste recycling and wastewater treatment.

## Supporting information

S1 File Supporting information 3–7. (DOCX)

## References

[1] Elshehy A, El-Safty S, Shenashen M, Reproducible Design for the Optical Screening and Sensing of Hg(II) Ions, Chemosensors 2014;2:219.

[2] Hussein MA, Alamry KA, Alsulami QA, Elshehy EA, El-Said WA. Design and synthesis of a combined meso-adsorbent/chemo-sensor for extraction and detection of silver ions. Spectrochim Acta A Mol Biomol Spectrosc. 2022;272:120938. doi: 10.1016/j.saa.2022.120938 35124483

[3] Alsulami Q, Hussein M, Alsheheri S, Elshehy EA, El-Said W. Unexpected ultrafast and high adsorption performance of Ag(I) and Hg(II) ions from multiple aqueous solutions using microporous functional silica-polymer sponge-like composite. J Mater Sci Technol. 2022;17:2000–13.

[4] Sun Z, Xiao Y, Sietsma J, Agterhuis H, Yang Y. A Cleaner Process for Selective Recovery of Valuable Metals from Electronic Waste of Complex Mixtures of End-of-Life Electronic Products. Environ Sci Technol. 2015;49(13):7981–8. doi: 10.1021/acs.est.5b01023 26061274

[5] Jia YF, Steele CJ, Hayward IP, Thomas KM. Mechanism of adsorption of gold and silver species on activated carbons. Carbon. 1998;36(9):1299–308. doi: 10.1016/s0008-6223(98)00091-8

[6] Cantuaria ML, de Neto AFA, Nascimento ES, Vieira MGA. Adsorption of silver from aqueous solution onto pre-treated bentonite clay: complete batch system evaluation. J Clean Prod. 2016;112:1112–21.

[7] Atia AA, Donia AM, Heniesh AM. Adsorption of silver and gold ions from their aqueous solutions using a magnetic chelating resin derived from a blend of bisthiourea/thiourea/glutaraldehyde. Sep Sci Technol. 2014;49:2039–48.

[8] Elshehy EA, Shenashen MA, El-Magied MOA, Tolan DA, El-Nahas AM, Halada K, et al. Selective recovery of silver (I) ions from e-waste using cubically-multi-thiolated cage mesoporous monoliths. Eur J Inorg Chem. 2017;2017:4823–33.

[9] El-Sawaf A, Tolan D, Abdelrahman M, El-Hay IA, Ismael M, Ahmed A, et al. Fast in-situ synthesis of mesoporous Prussian blue-silica nanocomposite for superior silver ions recovery performance. J Chem Technol Biotechnol. 2024;99:1941–54.

[10] Zhou X, Li Y, Liu J. Highly Efficient Removal of Silver-Containing Nanoparticles in Waters by Aged Iron Oxide Magnetic Particles. ACS Sustain Chem Eng. 2017;5(6):5468–76. doi: 10.1021/acssuschemeng.7b00797

[11] Freitas GR, Vieira MG, da Silva MG. Characterization and biosorption of silver by biomass waste from the alginate industry. J Clean Prod. 2020;271:122588.

[12] Hussein MA, Alamry KA, El Shishtawy RM, Elshehy EA, El-Said WA. Nanoporous colorant sensors and captors for simultaneous recognition and recovery of gold from E-wastes. Waste Manag. 2020;116:166–78. doi: 10.1016/j.wasman.2020.07.030 32799098

[13] Atia AA. Adsorption of silver(I) and gold(III) on resins derived from bisthiourea and application to retrieval of silver ions from processed photo films. Hydrometallurgy. 2005;80(1–2):98–106. doi: 10.1016/j.hydromet.2005.07.004

[14] Lotfi B, Tarlani A, Akbari-Moghaddam P, Mirza-Aghayan M, Peyghan AA, Muzart J, et al. Multivalent calix[4]arene-based fluorescent sensor for detecting silver ions in aqueous media and physiological environment. Biosens Bioelectron. 2017;90:290–7. doi: 10.1016/j.bios.2016.11.065 27931003

[15] Donia A, Atia A, Daher A, Desouky O, Elshehy EA. Synthesis of amine/thiol magnetic resin and study of its interaction with Zr(IV) and Hf(IV) ions in their aqueous solutions. J Disper Sci Technol. 2011;32:634.

[16] Celik Z, Gülfen M, Aydin AO. Synthesis of a novel dithiooxamide-formaldehyde resin and its application to the adsorption and separation of silver ions. J Hazard Mater. 2010;174(1–3):556–62. doi: 10.1016/j.jhazmat.2009.09.087 19819621

[17] Sakamoto H, Ishikawa J, Koike M, Doi K, Wada H. Adsorption and concentration of silver ion with polymer-supported polythiazaalkane resins. React Funct Polym. 2003;55(3):299–310.

[18] Asakawa T, Inoue K, Tanaka T. Adsorption of Silver on Dithiocarbamate Type of Chemically Modified Chitosan. Kagaku Kogaku Ronbunshu. 2000;26(3):321–6. doi: 10.1252/kakoronbunshu.26.321

[19] Ansari R, Delavar AF. Application of poly 3-methylthiophene for removal of silver ion from aqueous solutions. J Appl Polym Sci. 2009;113(4):2293–300.

[20] Tunçeli A, Türker AR. Flame atomic absorption spectrometric determination of silver after preconcentration on Amberlite XAD-16 resin from thiocyanate solution. Talanta. 2000;51(5):889–94. doi: 10.1016/s0039-9140(99)00348-3 18967920

[21] Tolan D, Heniesh A, Ismael M, Elshehy E, Alqahtani NF, El-Said WA, et al. Removal of Mercury Ions from Aqueous Solutions Using Dithiooxamide-Glutaraldehyde Resin. Solvent Ext Ion Exc. 2023;41(7):958–73. doi: 10.1080/07366299.2023.2259951

[22] Atia AA, Donia AM, Yousif AM. Synthesis of amine and thiol chelating resins and study of their interaction with zinc(II), cadmium(II) and mercury(II) ions in their aqueous solutions. React Funct Polym. 2003;56(1):75–82.

[23] El-Ghaffar MAA, Mohamed MH, Elwakeel KZ. Adsorption of silver(I) on synthetic chelating polymer derived from 3-amino-1,2,4-triazole-5-thiol and glutaraldehyde. Chem Eng J. 2009;151(1–3):30–8.

[24] Mehdaoui R, Agren S, Dhahri A, El Haskouri J, Beyou E, Lahcini M, et al. New sonochemical magnetite nanoparticles functionalization approach of dithiooxamide–formaldehyde developed cellulose: from easy synthesis to recyclable 4-nitrophenol reduction. Appli Organomet Chem. 2021;35(6):e6257.

[25] Shim AK, Seo KD, Kim HJ. Synthesis of MnS/MnO decorated N, S-doped carbon derived from a Mn(II)-coordinated polymer for the catalytic oxidation of H2O2 and bisphenol. Adv Funct Mater. 2023;33(18):2210549.

[26] Tolan DA, El-Sawaf AK, Alhindawy IG, Ismael MH, Nassar AA, El-Nahas AM, et al. Effect of bismuth doping on the crystal structure and photocatalytic activity of titanium oxide. RSC Adv. 2023;13(36):25081–92. doi: 10.1039/d3ra04034h 37622010 PMC10445215

[27] Althagafi II, Ahmed SA, El-Said WA. Colorimetric aflatoxins immunoassay by using silica nanoparticles decorated with gold nanoparticles. Spectrochim Acta A Mol Biomol Spectrosc. 2021;246:118999. doi: 10.1016/j.saa.2020.118999 33038860

[28] El-Said WA, Fouad DM, Ali MH, El-Gahami MA. Green synthesis of magnetic mesoporous silica nanocomposite and its adsorptive performance against organochlorine pesticides. Int J Environ Sci Technol. 2017;15(8):1731–44. doi: 10.1007/s13762-017-1530-9

[29] Parr RG, Yang W. Density-Functional Theory of Atoms and Molecules. New York: Oxford University Press; 1989.

[30] Ziegler T. Approximate density functional theory as a practical tool in molecular energetics and dynamics. Chem Rev. 1991;91(5):651–67.

[31] Frisch M, Trucks G, Schlegel H, Scuseria G, Robb M, Cheeseman J, et al. Gaussian Inc., Wallingford, CT, 2009;270:271.

[32] Becke AD. Density‐functional thermochemistry. III. The role of exact exchange. J Chem Phys. 1993;98:5648–52.

[33] Lee C, Yang W, Parr R. Development of the Colle-Salvetti correlation-energy formula into a functional of the electron density. Phys Rev B Condens Matter. 1988;37(2):785–9. doi: 10.1103/physrevb.37.785 9944570

[34] Stephens PJ, Devlin FJ, Chabalowski C, Frisch MJ. Ab initio calculation of vibrational absorption and circular dichroism spectra using density functional force fields. J Phys Chem. 1994;98:11623–7.

[35] Zhurko GA, Zhurko DA. Chemcraft. Version 1.7 (Build 132). Available from: www.chemcraftprog.com

[36] Song X, Gunawan P, Jiang R, Leong SSJ, Wang K, Xu R. Surface activated carbon nanospheres for fast adsorption of silver ions from aqueous solutions. J Hazard Mater. 2011;194:162–8. doi: 10.1016/j.jhazmat.2011.07.076 21862215

[37] Weber WJ, Morris JC. Kinetics of adsorpion on carbon from solutions. J Sanit Eng Div Am Soc Civ Eng. 1963;89:31–59.

[38] Alhindawy IG, Elshehy EA, El-Khouly ME, Abdel-Monem YK, Atrees MS. Fabrication of Mesoporous NaZrP Cation-Exchanger for U(VI) Ions Separation from Uranyl Leach Liquors. Colloids Interfaces. 2019;3(4):61. doi: 10.3390/colloids3040061

[39] Yousif A, Zaid O, El-Said W, Elshehy E, Ibrahim I, Silica N. Silica nanospheres coated nanofibrillated cellulose for removal and detection of copper(II) ions in aqueous solutions. Ind Eng Chem Res. 2019;58:4828–37.

[40] Tahir SS, Rauf N. Thermodynamic studies of Ni(II) adsorption onto bentonite from aqueous solution. J Chem Therm. 2003;35:2003–9.

[41] Langmuir I. The adsorption of gases on plane surfaces of glass, mica and platinum. J Chem Am Soc. 1918;40:1361–403.

[42] Freundlich HMF. Uber die adsorption in losungen. Z Phys Chem. 1906;57:387–470.

[43] Abd El-Ghaffar MA, Abdel-Wahab ZH, Elwakeel KZ. Extraction and separation studies of silver(I) and copper(II) from their aqueous solution using chemically modified melamine resins. Hydrometallurgy. 2009;96(1–2):27–34. doi: 10.1016/j.hydromet.2008.07.008

[44] Donia AM, Atia AA, Elwakeel KZ. Recovery of gold (III) and silver (I) on a chemically modified chitosan with magnetic properties. Hydrometallurgy. 2007;87:197–206.

[45] Donia AM, Atia AA, El-Boraey HA, Mabrouk DH. Adsorption of Ag(I) on glycidyl methacrylate/N,N-methylene bis-acrylamide chelating resins with embedded iron oxide. Sep Purif Technol. 2006;48:281–7.

[46] Wu K, Wang B, Tang B, Luan L, Xu W, Zhang B, et al. Adsorption of aqueous Cu(II) and Ag(I) by silica anchored Schiff base decorated polyamidoamine dendrimers: behavior and mechanism. Chin Chem Lett. 2022;33:2721–5.

[47] Długosz O, Banach M. Kinetic, isotherm and thermodynamic investigations of the adsorption of Ag and Cu2 on vermiculite. J Mol Liq. 2018;258:295–309.

[48] Freitas EDd, Almeida HJd, Neto AFdA, Vieira MGA. Continuous adsorption of silver and copper by Verde-lodo bentonite in a fixed bed flow-through column. J Clean Prod. 2018;171:613–21.

[49] Vicente-Martínez Y, Ruiz-Mendieta M, Caravaca-Garratón M, Hernández-Córdoba M, López-García I. Fast Procedure for Removing Silver Species in Waters Using a Simple Magnetic Nanomaterial. Separations. 2023;10(7):398. doi: 10.3390/separations10070398

[50] Huang Y, Wu Y, Ding W, Sun Q, Hu C, Liu B, et al. Anion-synergistic adsorption enhances the selective removal of silver ions from complex wastewater by chitosan-coated magnetic silica core-shell nanoparticles. J Clean Prod. 2022;329:130777.

[51] Islam MA, Parvin MI, Dada TK, Kumar R, Antunes E. Silver adsorption on biochar produced from spent coffee grounds: validation by kinetic and isothermal modelling. Biomass Conv Bioref. 2024;14:28007–21.

[52] Júnior WJDN, Landers R, da Silva MGC, Vieira MGA. Equilibrium and desorption studies of the competitive binary biosorption of silver(I) and copper(II) ions on brown algae waste. J Environ Chem Eng. 2021;9:104840.

[53] Shao P, Chang Z, Li M, Lu X, Jiang W, Zhang K, et al. Mixed-valence molybdenum oxide as a recyclable sorbent for silver removal and recovery from wastewater. Nat Commun. 2023;14(1):1365. doi: 10.1038/s41467-023-37143-2 36914674 PMC10011435

[54] Zhang B, Wang S, Fu L, Zhao J, Zhang L, Peng J. Selective adsorption of silver ions from highly acidic aqueous solutions by polymers containing multiple sulfur groups. Water Air Soil Pollut. 2018;229:199.

[55] Celik Z, Gülfen M, Aydin AO. Synthesis of a novel dithiooxamide-formaldehyde resin and its application to the adsorption and separation of silver ions. J Hazard Mater. 2010;174(1–3):556–62. doi: 10.1016/j.jhazmat.2009.09.087 19819621

[56] Li Q-Y, Wang L, Yu X, Xu L. Highly efficient removal of silver nanoparticles by sponge-like hierarchically porous thiourea-formaldehyde resin from Water. J Hazard Mater. 2020;400:123184. doi: 10.1016/j.jhazmat.2020.123184 32563908

[57] Liao W, Liu Y, Liu Y. Adsorption of Ag(Ⅰ) on chelate resins containing N and S: Comparison and computational study with anionic resin. Sep Purif Technol. 2024;339:126600.

[58] Li Y, Wang X, Xia J, Zhou G, Wang X, Wang D, et al. Flower-like Thiourea-Formaldehyde Resin Microspheres for the Adsorption of Silver Ions. Polymers (Basel). 2023;15(11):2423. doi: 10.3390/polym15112423 37299222 PMC10255821

[59] Hao Z, Guo Y, Wu P, Mansuer M, Zhu J. Adsorption properties of silver ions on thiourea-formaldehyde resin. Adv Mater Res. 2014;868:459–62.

[60] Wyttenbach T, Liu D, Bowers MT. Interactions of the hormone oxytocin with divalent metal ions. J Am Chem Soc. 2008;130(18):5993–6000. doi: 10.1021/ja8002342 18393501

[61] Singha Deb AK, Dwivedi V, Dasgupta K, Musharaf Ali Sk, Shenoy KT. Novel amidoamine functionalized multi-walled carbon nanotubes for removal of mercury(II) ions from wastewater: Combined experimental and density functional theoretical approach. Chem Eng J. 2017;313:899–911. doi: 10.1016/j.cej.2016.10.126

