# Supplementary material for: Efficient removal of silver ions from wastewater via chelation with dithiooxamide-functionalized polymeric adsorbent
Source: PLoS One. 2025 Dec 31;20(12):e0338510. doi: 10.1371/journal.pone.0338510 (PMC12755756; doi:10.1371/journal.pone.0338510)
Supplement: S1 File — (DOCX) [file pone.0338510.s001.docx]

**Supporting Information**

**Efficient Removal of Silver Ions from Wastewater via Chelation with Dithiooxamide-Functionalized Polymeric Adsorbent**

### Abdullah S. Al-Bogami^1^, Abdullah Akhdhar^1^, Dina A. Tolan^2,3^, Mohamed H. Ismael^4^, Emad A. Elshehy^4^, Waleed A. El-Said^1,*^

*^1^**College of Science, Department of Chemistry, University of Jeddah, P.O. Box 80327, Jeddah 21589, Saudi Arabia*

*^2^Department of Chemistry, College of Science and Humanities, Prince Sattam bin Abdulaziz University, Alkharj, 11942, Saudi Arabia*

*^3^Department of Chemistry, Faculty of Science, Menoufia University, Shibin El-Kom, Egypt*

*^4^ Nuclear Materials Authority, P.O. Box 530, El Maadi, Cairo, Egypt*

**
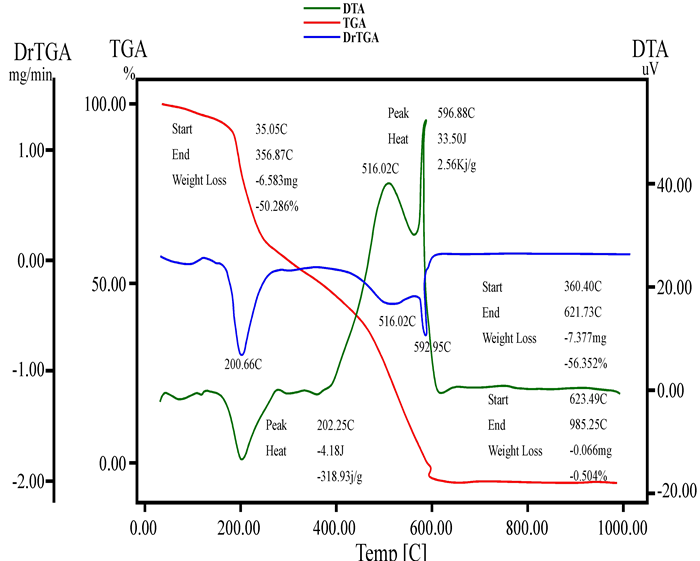
**

**Figure S1.** The TGA, DTG, and DSC analyses of the DTG-R.

**Table S1** Selected bond lengths (B, Ǻ) and NAO bond order (BO) for some selected complexes.

|  | Complexes | **Ag1-S1** | **Ag1-S2** | **Ag1-N4** | **Total BO for Ag** |
| --- | --- | --- | --- | --- | --- |
| B | **1** | 2.575 | 2.507 | - |  |
|  | **2** | 2.604 | - | 2.225 |  |
|  | **5** | 2.614 | 2.610 | - |  |
| BO | **1** | 0.7690 | 0.8607 | - | 1.6297 |
|  | **2** | 0.3550 | - | 0.2877 | 0.8587 |
|  | **5** | 0.3763 | 0.3642 | - | 0.7405 |

**Table S2**: NBA charges of the ligand (thione form) and the studied complexes at B3LYP/3-21 G(d).

| **atom/compd** | **L** | **1** | **2** | **3** | **4** | **5** |
| --- | --- | --- | --- | --- | --- | --- |
| **S1** | -0.151 | -0.227 | -0.242 | -0.226 | -0.392 | -0.139 |
| **S2** | -0.132 | -0.358 | -0.222 | -0.354 | -0.306 | 0.131 |
| **S3** | -0.144 | -0.141 | -0.148 | -0.223 | -0.149 | -0.122 |
| **S4** | -0.163 | -0.174 | -0.166 | -0.324 | -0.167 | -0.140 |
| **N1** | -0.553 | -0.530 | -0.535 | -0.533 | -0.682 | -0.521 |
| **N2** | -0.558 | -0.559 | -0.667 | -0.544 | -0.550 | -0.689 |
| **N3** | -0.551 | -0.547 | -0.601 | -0.413 | -0.555 | -0.664 |
| **N4** | -0.557 | -0.445 | -0.549 | -0.457 | -0.400 | -0.517 |
| **Ag1** | - | 0.573 | 0.632 | 0.575 | 0.645 | 0.714 |
| **Ag2** | - | - | - | 0.575 | 0.601 | - |
